# Addressing Specific (Poly)ion Effects for Layer-by-Layer Membranes

**DOI:** 10.1021/acsapm.2c02078

**Published:** 2023-02-10

**Authors:** Daniëlle Scheepers, Anna Casimiro, Zandrie Borneman, Kitty Nijmeijer

**Affiliations:** Membrane Materials and Processes, Department of Chemical Engineering and Chemistry, Eindhoven University of Technology, P.O. Box 513, Eindhoven 5600 MB, The Netherlands

**Keywords:** nanofiltration, layer-by-layer, specific ion effects, chaotropicity, polyelectrolyte

## Abstract

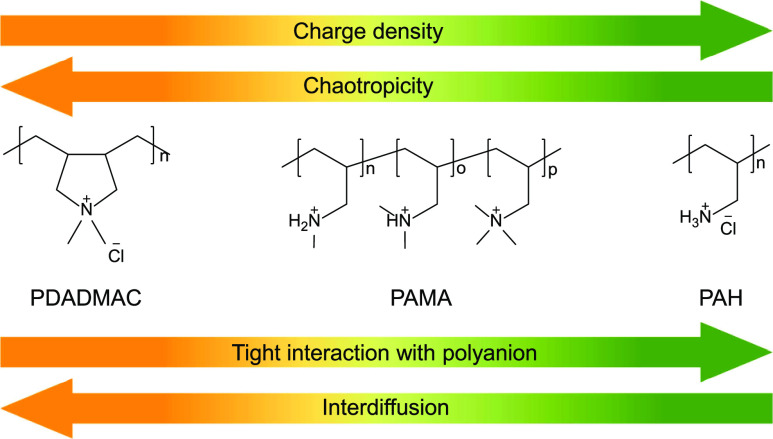

Layer-by-layer (LbL) assembly of the alternating adsorption of oppositely charged polyions is an extensively studied method to produce nanofiltration membranes. In this work, the concept of chaotropicity of the polycation and its counterion is introduced in the LbL field. In general, the more chaotropic a polyion, the lower its effective charge, charge availability, and hydrophilicity. Here, this is researched for the well-known PDADMAC (polydiallyldimethylammonium chloride) and PAH (poly(allylamine) hydrochloride), and the synthesized PAMA (polyallylmultimethylammonium), with two different counterions (I^–^ and Cl^–^). Higher chaotropicity (PDADMAC > PAMA-I > PAMA-Cl > PAH) translates into a reduced charge availability and a more pronounced extrinsic charge compensation, resulting in more mass adsorption and a higher pure water permeability. PAMA-containing membranes show the most interesting results in the series. Due to its molecular structure, the chaotropicity of this polycation perfectly lies between PDADMAC and PAH. Overall, the chaotropicity of PAMA membranes allows for the formation of the right balance between extrinsic and intrinsic charge compensation with PSS. Moreover, modifying the nature of the counterions of PAMA (I^–^ or Cl^–^) allows to tune the density of the multilayer and results in lower size exclusion abilities with PAMA-I compared to PAMA-Cl (higher MWCO and lower MgSO_4_ retention). In general, the contextualization of the polyion interaction within the specific (poly)ion effects expands the understanding of the influence of the charge density of polycations without ignoring the chemical nature of the functional groups in their monomer units.

## 1 Introduction

To meet increasing water demands, membrane separation technologies are one of the most promising and sustainable solutions.^1,2^ Commercial nanofiltration (NF) membranes have been used extensively to purify wastewater streams.^3−5^ Unfortunately, the production pathway for NF membranes commonly uses organic, toxic solvents.^6,7^

A much more sustainable alternative is the production of NF membranes via the layer-by-layer (LbL) method. In this method, oppositely charged water-soluble polyions, that is, polyelectrolytes (PEs) are adsorbed onto a charged porous support.^8^ One set of two PE layers is called one bilayer (BL) and consists of a positively charged and a negatively charged polyion layer, the polycation (PC) and polyanion (PA), respectively. Due to the oppositely charged nature of the PC and the PA, they can form ionic linkages between each other, resulting in the formation of the BLs. The dissociation of counterions present in the structure of the PE, and consequent release from the PE surface, is the main driving force for PE adsorption. This is due to the entropy gain that accompanies the release of the counterions upon assembly of the PE layers.^9,10^ The build-up of layers ultimately results in the formation of NF membranes with a high level of control over layer properties such as porosity, thickness, and chemical composition.^11−13^

Between the parameters explored in the preparation of LbL membranes, the charge density (CD) of PEs is frequently used to tune the multilayer structure and ultimately the membrane performance.^14−17^ In this context CD, defined as the number of charges per carbon atom in the monomer unit, essentially represents how much charge is stored in a PE.^18^ PEs with a high CD can form more ionic linkages than low-CD PEs. Due to this, PEs with a high CD will form thinner and denser layers compared to PEs with a low CD, thus impacting the membrane’s performance.^19,20^

As an example, two frequently used PCs in LbL membrane preparation are PDADMAC (poly diallyldimethylammonium chloride) and PAH (poly(allylamine) hydrochloride), in which the latter has a significantly higher CD than the former. These PCs are often combined with poly(sodium-4-styrenesulfonate) (PSS) as PA.^7,19,21−25^ Both combinations form NF membranes with good multivalent ion retention.^7,19^

Generally, the CD of PEs can be a good descriptor of the interaction of charged functional groups in the monomer unit with water. That is, PAH interacts better with water than PDADMAC due to its higher CD, higher charge availability (due to less steric hindrance of methyl groups), and higher hydrophilicity.^26^ This concept is confirmed by the hydration of differently substituted ammonium salts which increases with a decreasing number of substituents, so the PAH monomer unit is better hydrated than that of PDADMAC.^27^ The thermodynamic reason for this behavior is the entropy gain resulting from the formation of intrinsic ionic linkages between the oppositely charged PEs.^9,10^ The higher CD of PAH compared to PDADMAC means that in solution, more counterions are present in its surroundings for electroneutralization purposes. During the formation of the ionic linkages between PAH and PSS, the release of the aforementioned counterions happens and the amount of counterions released is higher for PAH than for PDADMAC causing a higher entropic gain for the formation of the PAH/PSS couple.^9^

This reasoning of entropic gain fails to explain why certain PEs with high CDs, do not form tight ion pairs.^28^ An interesting attempt in clarifying this behavior was done by Schlenoff et al. by introducing the concept of hydrophilicity of the PE couple.^29,30^ According to this concept, the formation of a strongly associated PE couple is well represented by the low water content of its PC/PA repeating unit. The lower the hydrophilicity of the PE couple (PC and PA together), the more tightly associated the PEs are. In this regard, the couple PAH/PSS shows a lower water content and higher strength of the PE association when compared to PDADMAC/PSS and it results in denser layers. Even though this is valid and succeeds in going beyond the commonly used CD concept, it is not supported by theoretical insights, and it lacks predictability limiting its range of applicability to already studied PE couples.

On the other hand, the interaction of PEs with their counterions and water can also be correlated to theories that address specific ion effects.^31−36^ Ions, including polyions, can be roughly sorted, based on their degree of hydration, into kosmotropes (highly hydrated) or chaotropes (poorly hydrated).^31,36^ More specifically, according to the law of matching water affinities (LMWA), polyions (hence, PEs) can be classified based on the size of their monomer unit, the amount of charge that they hold (e.g., their CD), combined with the specific chemical nature of the charged groups and its affinity with water.^32^ Therefore, chaotropicity and kosmotropicity include the accessibility of charges, CD, and hydrophobicity.^32,34,37,38^ Moreover, this influences the formation of ion pairs, as the LWMA indicates that kosmotropes preferentially pair with kosmotropes and chaotropes prefer chaotropes.^32,36^

In this regard, PDADMAC and PAH are both considered chaotropic, but the extent of their chaotropicity is different.^31,34,35,39^ The monomer unit of PDADMAC is constituted of quaternary ammonium moieties, which results in higher chaotropicity, compared to PAH, which consists solely of primary ammonium groups. Although the amount of charge their monomer unit carries is the same (assuming that PAH is fully charged) but their size is significantly different. This is directly connected to the CD, but while the latter sets its boundary to a merely physical quantity, the specific (poly)ion effect concept includes the chemical nature of the charged groups as a variable. Although essential, this was previously ignored in the LbL field.

**Figure 1:**
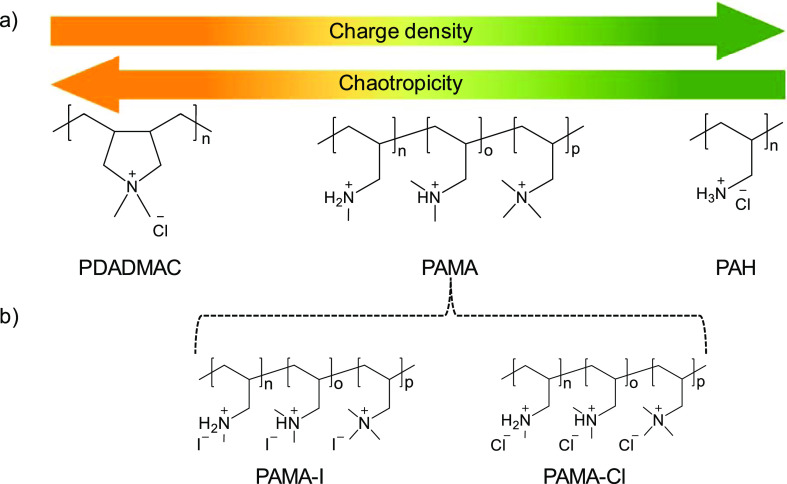
(a) Molecular structure of PDADMAC, PAMA, and PAH and their classification based on CD and chaotropicity; (b) molecular structure of PAMA-I and PAMA-Cl.

In this work, we now use the concept of LMWA and chaotropicity to investigate the influence of the PC used in the membrane fabrication process on the membrane’s performance. Limiting the study to the influence of the PC and its chaotropicity while keeping the PA similar, allows systematic evaluation, especially with the use of a chaotropic PA, like PSS, since chaotropes like to bind with chaotropes.^32,34,36^ This indicates that PSS is a good candidate for LbL formation with PDADMAC and PAH. Moreover, it expands the knowledge on the interactions between PCs and their counterions. Here, this is done by preparing LbL membranes using 3 different PCs with different chaotropicities and the same PA (PSS) for all of them. The PCs that are used are the well-known PDADMAC and PAH. In addition, poly(allylmultimethylammonium) (PAMA) was synthesized. The differences in structure, CD, and chaotropicity between these three PCs are given in Figure 1a.

As previously mentioned, the extent of chaotropicity of PDADMAC and PAH is different. Membranes prepared with the PAH/PSS couple show a high degree of association of the PEs, making this a good choice for multilayer formation.^19,40^ The high degree of association is a result of the high accessibility of charges with PAH. At the same time, in LbL membranes, these PEs give rise to relatively low water permeabilities, which is obviously undesired. On the other hand, although the PDADMAC/PSS couple delivers membranes with better permeabilities, their performances are highly dependent on the nature of the terminating layer, resulting in extreme differences in retention behaviors.^41^

PAMA is a PAH derivative (partially) consisting of quaternary ammonium ions (Figure 1a) that in terms of CD and chaotropicity perfectly lays in between those of PDADMAC and PAH, making it an interesting PE for membrane preparation when good rejection (like PAH) and improved water permeability (like PDADMAC) could be combined. PAMA as a fully methylated derivative was already synthesized by Wytrwal et al. for use in biotechnological applications but has not yet been used for the preparation of LbL membranes.^42^

In this work, we thus investigate the effect of the chaotropicity of the PC and correlate this to the actual membrane performance. Moreover, we also include the effect of the chaotropicity of the counterion of the PC. The use of different ions with a different chaotropic character has been studied frequently, and can significantly alter the LbL characteristics both on a structural level and on a performance one in NF membranes.^43−45^ In our work, the use of two different chaotropic counterions (I^–^ and Cl^–^) is employed to study the differences arising between the LbL membranes prepared in the presence of these counterions. Even though I^–^ and Cl^–^ are expected to give rise to similar interactions with the PE and with water, their different chaotropicity can have an impact on the membrane properties and performances.

Therefore, this work consists of the preparation of LbL membranes using different PCs with different chaotropicity (PDADMAC, PAMA-I (Figure 1b), PAMA-Cl (Figure 1b), PAH). For all combinations, the PE adsorption and the surface charge of the membrane are investigated. Moreover, the membrane performance is quantified by measuring the pure water permeability (PWP), the molecular weight cut-off (MWCO), and several salt retentions.

## 2 Experimental Section

### 2.1 Materials

Two PCs were purchased: PDADMAC (*M*_w_ = <100 kDa, 20 wt % in water) from Sigma-Aldrich (The Netherlands) and PAH (*M*_w_ = 150 kDa) purchased from TCI chemicals (Belgium). As PA poly(sodium 4-styrene sulfonate) (PSS, *M*_w_ = 500–700 kDa, 20.4 wt % in water) from Tosoh Organic Chemical Co. LTD (Japan) was used. NaOH, NaI, D_2_O, DMSO, DMF, and THF were obtained from Sigma-Aldrich (The Netherlands). NMP was supplied by VIVoChem (The Netherlands).

Potassium chloride (KCl, *M*_w_ = 74.55 g/mol), magnesium sulfate heptahydrate (MgSO_4_·7H_2_O, *M*_w_ = 246.47 g/mol), magnesium chloride hexahydrate (MgCl_2_·6H_2_O, *M*_w_ = 203.30 g/mol), and ethanol absolute (>99.9% purity) were obtained from VWR Chemicals (Belgium). Sodium sulfate decahydrate (Na_2_SO_4_·10H_2_O, *M*_w_ = 322.20 g/mol) was obtained from Acros Organics (Belgium). Sodium chloride (NaCl, *M*_w_ = 58.44 g/mol) was obtained from Nouryon (SanalP, pharmaceutical grade) (The Netherlands). Polyethylene glycols (PEGs) with different molecular weights (*M*_w_ = 200, 400, 600, 1500, and 4000 g/mol) were obtained from Sigma-Aldrich (The Netherlands). All chemicals were used as supplied.

### 2.2 Characterization Methods

Nuclear magnetic resonance spectroscopy (NMR) spectra were recorded at room temperature on a Bruker, FT-NMR spectrometer AVANCE III HD-Nanobay (400 MHz, Bruker Ultrashield magnet, BBFO Probehead, BOSS1 shim assembly) in D_2_O (Sigma-Aldrich). Chemical shifts are given in ppm.

Elemental analysis was performed by Mikrolab Kolbe (Oberhausen, Germany).

### 2.3 Synthesis of PAMA

#### 2.3.1 Synthesis of PAMA-I

The synthesis of the PAMA-I was performed by modifying a procedure reported in the literature.^42^ In a round bottom flask, PAH (3 g) was added and dissolved in 24 mL of water under stirring at room temperature. After observing the complete dissolution of the solids, NaOH (1.59 g, 47 mmol) was slowly added while stirring. The system was left reacting for 10 min. After this time, NMP (150 mL) was added to the reaction flask and left stirring for 30 min. NaI (9 g, 60 mmol) and 30 mL of CH_3_I were added, and the system was left reacting at 50 °C for 2 days under a nitrogen flow while stirring. After this time, the reaction flask was allowed to cool down to room temperature, and the mixture was precipitated in THF (50 mL). The system was decanted, and the solids were collected and washed with fresh THF. The solids were then dissolved in water (∼50 mL), and the solution thus obtained was poured into two dialysis tubes. The dialysis tubes (MWCO 2 or 50 kDa) were obtained from Spectra/Por. The system was left dialyzing in water (5 L per tube) for 3 days, refreshing the water every day. The purified products were then collected in a round bottom flask, and the excess water was removed by rotary evaporation. The orange polymer thus obtained was then dried in a vacuum oven at 50 °C overnight.

#### 2.3.2 Synthesis of PAMA-Cl

PAMA-I (1 g) was dissolved in 50 mL of water, and the solution obtained was placed in a dialysis tube. The tube was left dialyzing in KCl (0.1 M) for 3 days refreshing the solution every day, followed by 3 days in water. The excess solvent was removed by rotary evaporation, and the orange solid obtained was dried in a vacuum at 50 °C overnight.

### 2.4 Polyelectrolyte Adsorption

Fixed-angle optical reflectometry was used to study the alternating adsorption of the PEs onto a flat, reflective silica wafer. The reflectometer setup is custom-made by Wageningen University & Research (The Netherlands). Using a stagnation point flow cell, the PE solution was flown to a silicon wafer with a silica layer on top until a stable adsorption plateau was reached. PE solutions containing the PC or PA (0.01 wt % PE) with ionic strengths of 0.05 M NaCl were prepared and alternately adsorbed at room temperature onto the wafer. After each PE adsorption step, the wafer was rinsed using a 0.05 M NaCl solution. Previously reported optical reflectometry measurements confirm that such measurements are highly reproducable^46,47^ with error bars of less than 5%. For this reason, after confirming the reproducibility for PAH/PSS for our experimental setup again, the optical reflectometry measurements were performed only once for each sample.

Here, polarized monochromatic light (He/Ne laser 1108P, 632.8 nm, power < 1 mW) hit the wafer at approximately the Brewster angle and was reflected toward a detector. This reflected light was split into parallel (p) and perpendicular (s) polarized components, and the ratio between the change in these components, that is, Δ*S*, was directly proportional to the mass adsorbed onto the wafer, as shown in eq 1.
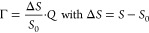
1

Γ is the amount of mass adsorbed onto the wafer (mg/m^2^). *S*_0_ is the initial output signal of the bare silicon wafer (−), where *S* is defined as the ratio between the intensities of the two components p (parallel) and s (perpendicular), and finally, *Q* is the sensitivity factor. This factor is dependent on the configuration of the setup (calculated using the software program Prof. Huygens, version 1.3).^48^

### 2.5 Membrane Preparation

#### 2.5.1 Membrane Support

Hollow fiber tight ultrafiltration inside-out membranes with a MWCO value of 10 kDa were kindly provided by Pentair X-Flow (The Netherlands) and used as support for LbL deposition. The hollow fiber support consists of modified and unmodified polyethersulfone (PES). The hollow fibers have a length of 154 cm and an inner diameter of 0.8 mm and before coating, they were cut into lengths of 30 cm.^49^

#### 2.5.2 LbL membrane Preparation

Prior to coating the PE layers, the hollow fiber support was rinsed in an ethanol/water mixture (18 wt %) overnight to remove impurities. During coating, the support was fully immersed in the PE solution (0.01 wt % PE in 0.05 M NaCl) for 15 min with a refreshment every 5 min. The PC layer was coated first, and after that, the membranes were immersed in a 0.05 M NaCl rinsing solution for 15 min to remove any weakly bound PE present. This was followed by a PA layer, and this approach was repeated until the required number of BLs was applied. All PE solutions were used with an unmodified pH.

The PE combinations used to prepare the membranes were PDADMAC/PSS, PAMA-I/PSS, PAMA-Cl/PSS, and PAH/PSS. The PEs were coated for a total of nine BLs. After each subsequent PE layer coating, a sample of seven membranes was taken: one sample for surface charge characterization and six samples for the PWP and retention measurements. This was conducted for PC as well as PA-terminated membranes.

### 2.6 Membrane Charge

The surface charge of the LbL-coated membranes was determined using an electrokinetic analyzer (SurPASS 3, Anton Paar, Austria). The apparent zeta potential was derived from the streaming current generated by flowing an electrolyte solution along the membrane. The electrolyte solution consisted of a 5 mM KCl solution. Two membrane samples per layer were measured for 10 measurement cycles at room temperature at a constant pH of 5.8.

### 2.7 Membrane Performance

The performance of the prepared membrane sets in cross-flow operation was characterized by PWP, MWCO, and retention of 5 mM MgSO_4_, MgCl_2_, Na_2_SO_4,_ or NaCl solutions. The membranes were fixated with chromatographic connectors (Inacom Instruments, The Netherlands), and the feed was pressurized to obtain a transmembrane pressure of 3 bar with the use of a diaphragm pump (KNF, Switzerland), with a nominal flow of 6 L/min at atmospheric pressure. PWP and salt retention were calculated as described in our previous work.^14^

The MWCO was determined by measuring the lowest *M*_w_ of PEG at which 90% retention was observed. The concentration of each PEG in the feed solution was 1 g/L. The PEG concentration in the permeate and retentate samples was analyzed with gel permeation chromatography (GPC) (Shimadzu LC-2050C 3D series) and a size exclusion column (Shodex OHpak SB-802.5 HQ 8 × 300 mm^2^ column 200 Å, 6 μm). The flow rate was 1 mL/min, and the eluent was ultrapure water.

## 3 Results and Discussion

### 3.1 PAMA Polymer Characterization

**Figure 2:**
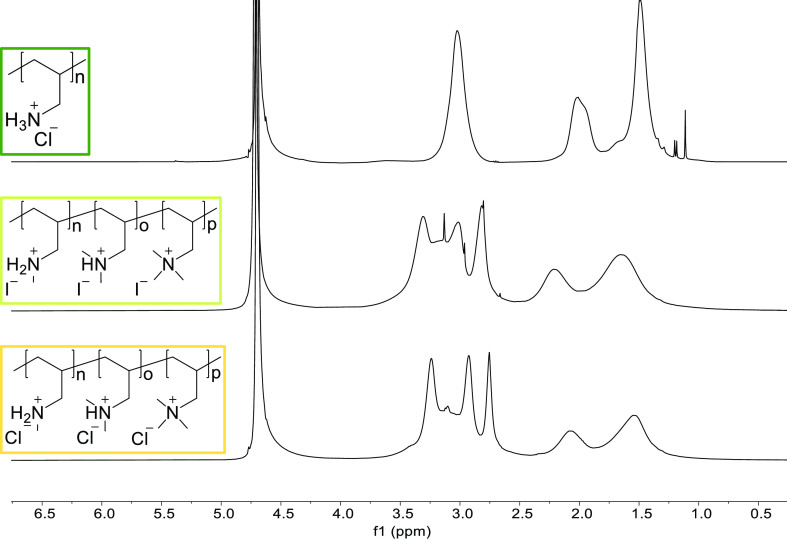
^1^H NMR spectra in D_2_O of PAH (top), PAMA-I (middle), and PAMA-Cl (bottom).

The successful methylation of PAH was confirmed via ^1^H NMR spectroscopy (Figure 2).

PAMA shows the presence of three signals attributed to different methylation degrees, indicating the formation of secondary, tertiary, and quaternary ammonium ions. A full quaternization is not obtained because of the high MW of the starting PAH used in our work (150 kDa), as the high MW limits the accessibility of the reactive polymer sites, impeding its complete quaternization.^50^ It is also known that the solvent chosen for the quaternization reaction (NMP) can hydrolyze in the presence of alkaline reagents and temperature.^51^ This decreases the neutralization of the charges of PAH and could give rise to side reactions between the product of the hydrolysis on NMP and the methylation reagent used in our work (methyl iodide). The integration of the peaks to quantify the methylation degree of the prepared polymers is not reported given the strong overlap of the peaks, hence the unreliability of the method.

Additionally, the substitution of iodide counterions with chloride counterions, hence the conversion of PAMA-I into PAMA-Cl, is not detectable with ^1^H NMR (Figure 2 middle and bottom). Therefore, the complete conversion is checked via elemental analysis (Table 1). Here, the theoretical percentage for an equal methylation degree between secondary, tertiary, and quaternary is calculated for PAMA-I and PAMA-Cl and compared with the measured weight percentage.

**Table 1:** Elemental Analysis (wt %) Results for PAMA-I and PAMA-Cl

		element (wt %)					
polymer		C	N	I	Cl	H	total
PAMA-I	calculated	28.23	6.58	59.65		5.68	100
	measured	28.41	6.52	59.21		5.71	99.85
PAMA-Cl	calculated	49.52	11.55		29.23	9.95	100
	measured	42.23	12.19	4.84	29.54	10.17	98.87

For PAMA-I as well as PAMA-Cl, the measured weight percentages are close to 100%, indicating high purity of the prepared polymers. Moreover, the calculated and measured weight percentages are very similar, confirming the reliability of the methylation degree as hypothesized from ^1^H NMR. For PAMA-Cl, the percentages of iodide (<5 wt %) show a 95% conversion of PAMA-I into PAMA-Cl.

### 3.2 Optical Fixed Angle Reflectometry

**Figure 3:**
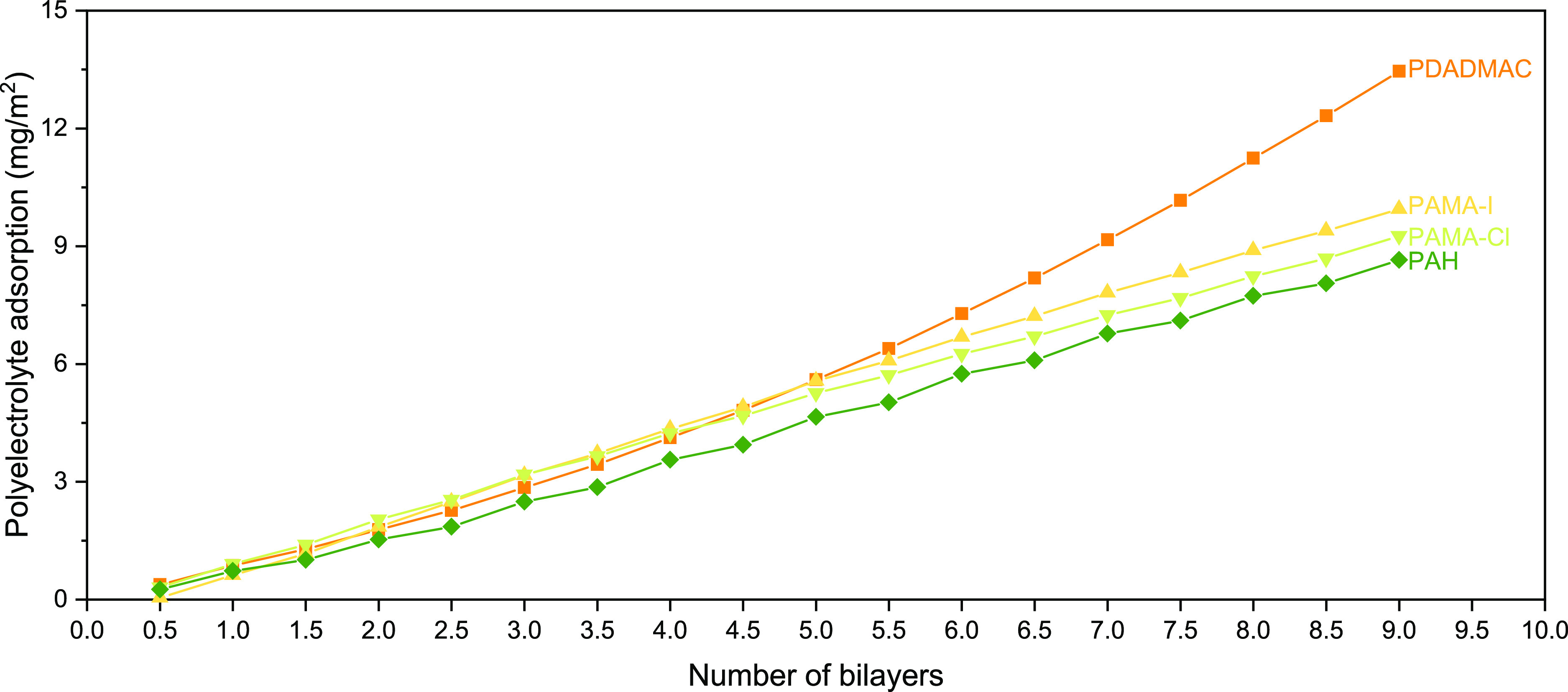
Adsorption of polyelectrolyte layers on silicon wafers monitored via optical reflectometry with four different PCs with decreasing chaotropicity: PDADMAC, PAMA-I, PAMA-Cl, and PAH paired with PSS. A PE concentration of 0.01 wt % and an ionic strength of the coating solution of 0.05 M NaCl are used.

To study the adsorption of the PEs onto a charged surface, optical fixed-angle reflectometry experiments were conducted (Figure 3). For all combinations, successful adsorption with every coating step is observed, as can be seen by the continuous increase of mass adsorption with every layer.

As seen in Figure 3, the mass adsorption of the PCs studied in this work decreases with decreasing chaotropicity: PDADMAC/PSS > PAMA-I/PSS > PAMA-Cl/PSS > PAH/PSS (Figure 1a). As mentioned before, an increase in chaotropicity signifies a decrease in the CD of the PCs as well as a decrease in charge availability. It must be acknowledged that the chaotropicity of the PC (and its charge availability) is not only defining the adsorption behavior because of the CD of the PE but also because of its interactions with its counterions. As previously discussed, according to the LMWA, the formation of tight ion pairs between ions of opposite charge and similar hydration degree (two chaotropes) is favorable. This can also be applied to the PCs studied in this work by scrutinizing the interactions of the PCs with their counterions.

In the case of PAH, its chaotropic character is the least pronounced among the PCs studied. We speculate that this defines the formation of preferential interactions with PSS (intrinsic linkages) rather than with Cl^–^ (its counterion), as the former is entropically more favorable.^9^ The formation of these intrinsic linkages results in low mass adsorption because the overcompensation of the charges necessary for LbL build-up is easily achieved. This is especially clear when PAH is compared with the highly chaotropic PDADMAC, which shows the opposite behavior. PDADMAC forms a relatively higher amount of extrinsic linkages, resulting in higher mass adsorption as necessary for charge overcompensation.

PAMA lays in between PAH and PDADMAC in terms of chaotropicity as well as CD, which is also seen in its mass adsorption. Two cases are distinguishable: the one in which the counterion is I^–^ and the one in which is Cl^–^. From the theory about specific ion effects, it is known that I^–^ is more chaotropic than Cl^–^^52^ and for this reason, it tends to form tight ion pairs with chaotropic cations. Translating this into the counterion-PC interactions implies that I^–^ forms more favorable interactions with PAMA than Cl^–^ does, as has been observed before for PDADMAC.^53^ The coating solution for PAMA-I, as well as PAMA-Cl, is 0.05 M of NaCl, making these the predominantly present ions. Therefore, it cannot be fully excluded that PAMA-I also interacts with the Cl^–^ ions present in the coating solution. However, in a solution with more than one chaotropic anion, the accumulation of the anion with a stronger chaotropic character (I^–^ in this case) at the PAMA surface is dominant.^36,54^ This results in more extrinsic charge compensation and thereby coiling of the PC chain. Coiling of PEs results in higher PE adsorption, as the PE charges are less accessible to compensate for the underlying adsorbed charges of the oppositely charged PE layer. This ultimately results in higher mass adsorption of PAMA-I than PAMA-Cl, as seen with reflectometry.

### 3.3 Zeta Potential

**Figure 4:**
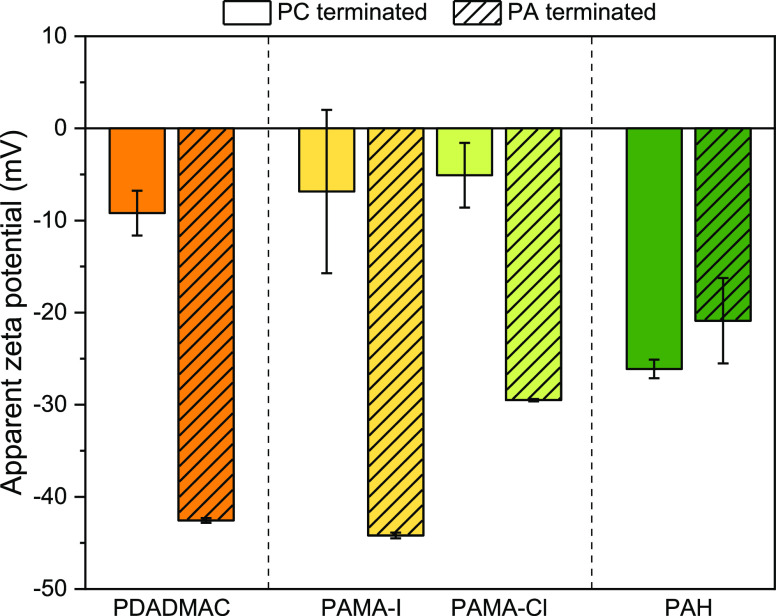
Influence of the polyion chaotropicity on the apparent zeta potential measured at pH 5.8 for PC and PA-terminated membranes (8.5 and 9 BLs), with decreasing chaotropicity of the PC (PDADMAC > PAMA-I > PAMA-Cl > PAH) in combination with PSS.

To evaluate the specific (poly)ion effect of the different PEs and counterions used during coating on the surface charge of the membranes prepared, zeta potential measurements were performed (Figure 4). The trends seen with zeta potential measurements do not reveal absolute values but provide information that allows us to rank the different PEs based on their relative surface charge.

As expected, most membranes are less negatively charged when they are terminated with a PC, compared to a PA layer. The addition of a PA layer results in the addition of negative charges on the membrane surface, hence the zeta potential decreases. The PAH/PSS membranes are an exception, but their values vary within the error range, making strong conclusions difficult.

Additionally, increasing the chaotropicity results in increasing differences between the PC (BL 8.5) and PA (BL 9) terminated membranes (so-called odd–even effects). Where PDADMAC membranes show a significant odd–even effect with the PC-terminated layer having a zeta potential almost seven times higher than the PA-terminated layer. In comparison, the odd–even effect is less extreme for all the other membranes prepared in this work. The reason for this is the significantly higher accumulation of PE per every layer adsorbed for PDADMAC/PSS, as seen before by optical reflectometry (Figure 3). Consequently, the charged nature of the different layers deposited (PC and PA) is well defined, and the differences observed with zeta potential measurements reflect this.

On the other hand, the lower chaotropicity of PAMA and PAH leads to the formation of thinner layers and, despite still showing clear odd–even effects, results in a less clear differentiation of the surface charge of the multilayer when compared to PDADMAC/PSS membranes.

### 3.4 Performance Measurement

#### 3.4.1 Pure Water Permeability

**Figure 5:**
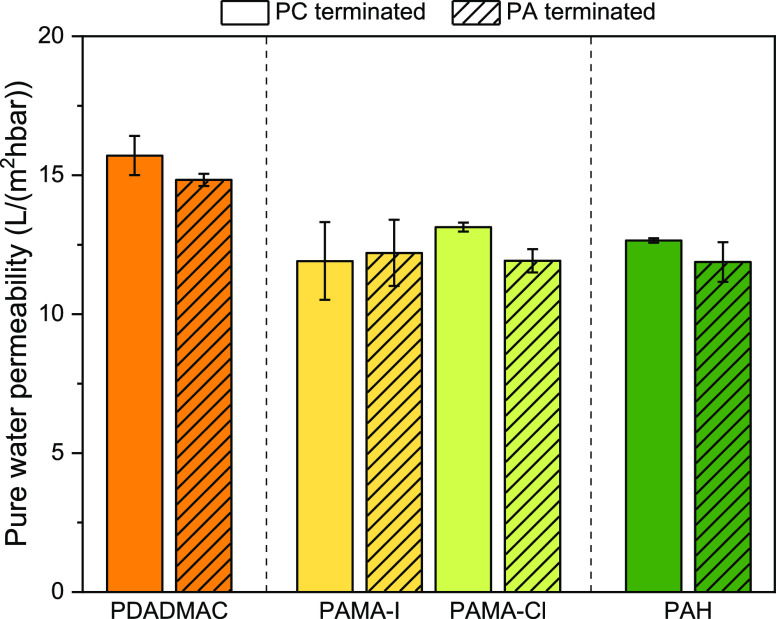
Influence of the polyion chaotropicity on the PWP for PC and PA-terminated membranes (8.5 and 9 BLs) with decreasing chaotropicity of the PC (PDADMAC > PAMA-I > PAMA-Cl > PAH) in combination with PSS.

PWP measurements were carried out to evaluate the impact of different counterions of the PCs and the chaotropicity of the PCs on the membrane performance (Figure 5).

The use of different PCs with increasing chaotropic character (and decreasing CD) influences the water permeability performances of the membranes prepared in this work. As seen in Figure 5, the membrane with the highest water permeability is clearly the one containing PDADMAC, which has the strongest chaotropic character, followed by PAMA and PAH, which show similar water permeabilities.

This order is in line with the reflectometry results (Figure 3). The PE couple that shows the highest mass adsorption is PDADMAC/PSS. As previously discussed, the chaotropicity of PDADMAC prevents it from exclusively forming tight intrinsic ionic linkages with PSS. Moreover, the presence of Cl^–^ as a counterion shields its charges and, together with water, acts as a plasticizer.^55^ These effects allow the PE couple PDADMAC/PSS to form more open layers than the other PE couples, and this translates into higher free volume and consequently higher PWP.

The membranes prepared with PAMA or PAH as PC have similar pure water permeabilities. The lower chaotropicity (and higher CD) of these PCs compared to PDADMAC, results in less PC adsorption to compensate for the previously deposited negative charges. However, the CD does not directly influence the number of intrinsic linkages. The lower permeability of PAMA and PAH is a clear indication of the influence of the type of charge on the formation of intrinsic linkages. In this case, PAMA and PAH are more effective in forming intrinsic ionic linkages with the PA compared to PDADMAC due to their less chaotropic nature. This results in less affinity with their natural counterion, thus more intrinsic charge compensation; hence, the extent of swelling is lower compared to PDADMAC, leading to thinner but denser multilayers and lower water permeabilities.

The chaotropicity of the counterion of PAMA (I^–^ or Cl^–^) does not significantly impact the water permeability of the prepared membranes. However, at this stage, it is impossible to distinguish if this is attributed to a similar chaotropic nature of the counterions employed (I^–^ and Cl^–^), or because of a balanced interplay between, on the one hand, increased membrane resistance and, on the other hand, increased openness of the layers. These effects could counteract each other, explaining the similar water permeabilities found for PAMA-I and PAMA-Cl.

#### 3.4.2 MWCO

**Figure 6:**
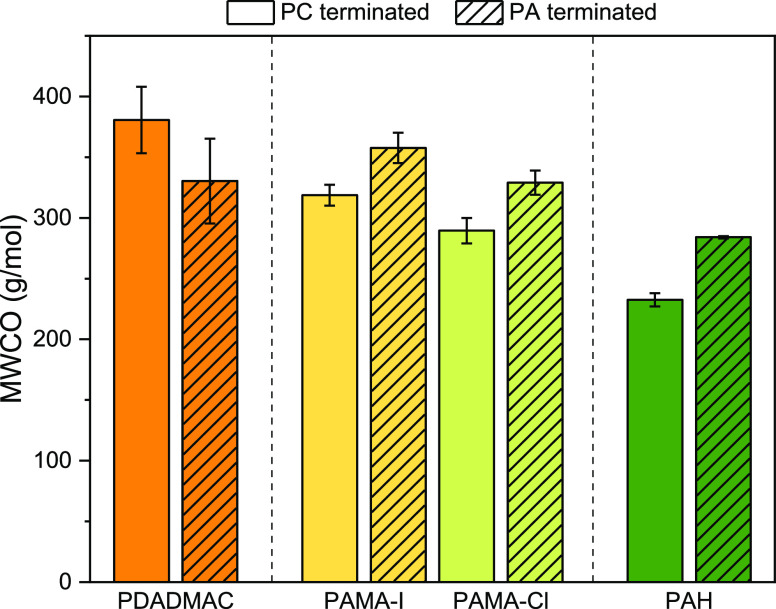
Influence of the polyion chaotropicity on the MWCO for PC and PA-terminated membranes (8.5 and 9 BLs), with decreasing chaotropicity of the PC (PDADMAC > PAMA-I > PAMA-Cl > PAH) in combination with PSS.

To evaluate the effect of the chaotropicity of the PCs and counterions used in this work on the size exclusion characteristics of the membranes prepared, MWCO measurements were performed (Figure 6).

Figure 6 shows that all the membranes prepared have an MWCO below 380 g/mol, meaning that they all have retention properties in the NF regime. Moreover, the size exclusion abilities of all membranes prepared are directly related to the PC’s chaotropicity. That is, the most chaotropic PC (PDADMAC) has the highest MWCO because PDADMAC is more subject to extrinsic charge compensation, is more coiled and forms the weakest ionic linkages. The least chaotropic PC (PAH) forms the tightest ionic linkages and retains the smallest uncharged solutes.^29,30^

In between those extremes of PDADMAC and PAH, as expected, lays PAMA which very clearly confirms that the chaotropicity of the PC not only influences the structural characteristics of the membranes but also their retention ability. Most importantly, the influence of the counterion is clearly visible in the retention behavior of the PAMA membranes. Indeed PAMA-I has on average a higher MWCO than PAMA-Cl regardless of the entity of the terminating layer (PC or PA). This is a confirmation that the interaction of I^–^ with PAMA is stronger than that with Cl^–^ leading to the formation of more open layers and a higher MWCO. As previously mentioned, the higher plasticizing effect of I^–^ on the PC delivers more open multilayers with lower size exclusion capabilities.

The odd–even trends observed in Figure 6 are peculiar and informative as well. For PDADMAC-containing membranes, PC-terminated (odd) layers retain bigger solutes than PA-terminated (even) ones. This is in line with the water permeability data in which PC-terminated membranes show higher water permeabilities and thus have more open layers than PA-terminated membranes. For the less chaotropic PCs (PAMA and PAH) the trend is the opposite, PC-terminated membranes show higher retentions for smaller solutes compared to PA-terminated ones. This different trend compared to PDADMAC is ascribed to the excess of positive charges for PAH and PAMA when PSS is adsorbed onto the multilayer. The free ammonium groups in PAH and PAMA protonate in the multilayer protone creating an excess of positive charge, which results in swelling for PSS-terminated membranes.^56^ Moreover, PAMA/PSS and PAH/PSS forms tight ionic linkages once complexed. Since PSS has a lower CD than PAMA and PAH, more PA needs to adsorb to compensate for the PC charges. This leads to more open structures for the PA layer than for the PC layer, resulting in the retention of smaller solutes for the PC-terminated membranes.

#### 3.4.3 Salt Retention

**Figure 7:**
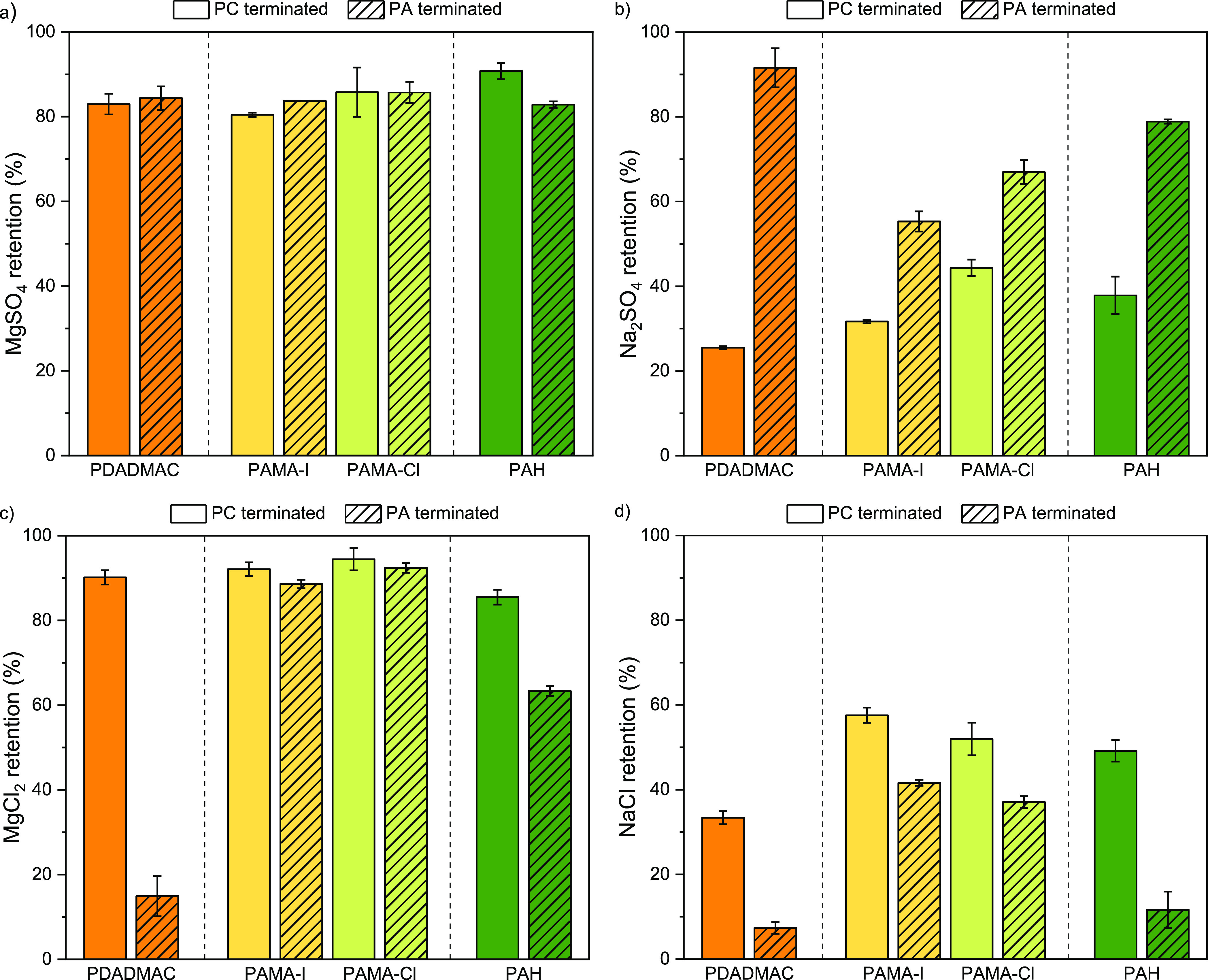
Influence of the polyion chaotropicity on the salt retention for PC and PA-terminated membranes (8.5 and 9 BLs), with decreasing chaotropicity of the PC (PDADMAC > PAMA-I > PAMA-Cl > PAH) in combination with PSS. (a) MgSO_4_ (b) Na_2_SO_4_ (c) MgCl_2_ (d) NaCl retention.

Salt retention measurements were carried out to evaluate the impact of specific (poly)ion effects and chaotropicity on the performance of the LbL membranes prepared (Figure 7).

Figure 7a shows that the MgSO_4_ retention is very similar for all membranes. This is surprising since the size exclusion capabilities of the membranes (MWCO Figure 6), do show significant differences charge exclusion is a crucial mechanism when charged ions are involved, as ions are significantly smaller than PEGs, so retention based on size is less likely for NF membranes.

Besides charge exclusion, it is important to acknowledge that the MgSO_4_ retention increases with decreasing chaotropicity of the PC employed. PAH-containing membranes show indeed the highest MgSO_4_ retention as the density of the multilayer prepared with this PC is the highest. Furthermore, the effect of chaotropicity is again also visible in the odd–even effects. PAH/PSS membranes show the opposite odd–even behavior as PDADMAC/PSS membranes. This arises from the significantly higher CD of PAH compared to PDADMAC, which results in higher intrinsic charge compensation of the former whereas the latter is more coiled, which leads to a more open terminating layer.

The retention of Na_2_SO_4_ (Figure 7b) and MgCl_2_ (Figure 7c) gives further insights into the charge exclusion character of the membranes prepared with different chaotropic PCs (PDADMAC, PAMA, and PAH) and the influence of the terminating layer. Clearly, for all membranes, the Na_2_SO_4_ retention is higher for BL 9 than for BL 8.5, and vice versa for MgCl_2_, as expected for more negatively charged membranes. However, the extent of the odd–even effects is significantly different between the prepared membranes. These effects are a result of PE layer build-up and an interplay of multilayer thickness and interdiffusion of the PEs between the layers. The thicker layers obtained with the adsorption of the most chaotropic PDADMAC result in more distinct odd–even effects. A more chaotropic character of a polyion results in more interaction with its respective counterion (extrinsic charge compensation). Extrinsic charge compensation limits the effective charge of the polyion, resulting in higher polyelectrolyte adsorption to overcompensate for the charges already present on the surface. Moreover, PCs with a lower CD (hence higher chaotropicity) have been shown to interdiffuse faster, because of weaker ionic pairing^57^ which normally results in larger odd–even effects. Therefore, PDADMAC/PSS shows the strongest odd–even effects, with BL 8.5 having the lowest Na_2_SO_4_ retention of all the membranes prepared. PDADMAC-containing membranes have the highest water permeability and the highest MWCO, indicating that they have the lowest size exclusion abilities. Moreover, given the fact that BL 8.5 is PC-terminated, this results in a relatively positive nature of the membrane surface charge, which negatively contributes to the retention of Na_2_SO_4_. For BL 9, this trend is switched: PDADMAC membranes show the highest Na_2_SO_4_ retention. This is caused by the extreme difference in the charged nature of the terminating PE as given by the zeta potential (Figure 4). However, as mentioned before, the trends seen with zeta potential measurements do not reveal absolute values as they are negative, whereas the PDADMAC-terminated membrane is clearly positive.

PAH is the least chaotropic PC investigated, resulting in the formation of the tightest ionic linkages thus, strong intrinsic charge compensation and low PE adsorption with limited interdiffusion between the layers. This results in some odd–even effects visible for Na_2_SO_4_ and MgCl_2_, but due to the small thickness of the layers, this is only limited compared to the PDADMAC/PSS membranes. Moreover, for PAH/PSS, when PSS is adsorbed onto the multilayer, the PAH in the membrane layers is known to protonate, creating an excess of positive charge.^56^ This is seen by the relatively little decrease in MgCl_2_ retention caused by the addition of the PSS layer (BL 9).

Lastly, from the results obtained with PAMA-I and PAMA-Cl, the influence of both the layer thickness as well as PE interdiffusion becomes apparent. Although PAMA-coated membranes have higher PE adsorption than PAH, they also have more interdiffusion between the layers. Those effects counteract each other and result in very limited differences in the retentions between odd and even BLs despite the significant difference in their charged nature, as observed from the zeta potential measurements (Figure 4). Moreover, as PAMA is a combination of strong quaternary ammonium groups compared with weak secondary and tertiary groups, the weak groups can exhibit the same behavior as for PAH. Additionally, due to the higher chaotropicity (implicating higher charge availability) of PAMA compared to PAH, this PC has more extrinsic charge compensation. These unbound, non-intrinsically linked groups are the groups responsible for the excess of positive charges, making it evident that PAMA has more excess of positive charges compared to PAH. This results in the highest MgCl_2_ retentions of the membranes prepared in this work and stable performances regardless of the nature of the terminating layer.

The nature of this positive charge is explained by a delicate balance between the CD of the PC and its interaction with the counterions. Given the chaotropic character of PAMA, it is hypothesized that the interaction with its counterions (I^–^ or Cl^–^) shields the charge effectively, allowing for the deposition of an excess of this PE. This is proven by the difference between PAMA-I and PAMA-Cl: PAMA-I has a higher adsorption due to more extrinsic charge compensation, which makes the excess of positive charges higher. This is not only seen in the lower Na_2_SO_4_ retention but also in the higher NaCl retention (Figure 7d), as it is easier to retain NaCl based on Na^+^ compared to Cl^–^.^27^

## 4 Conclusions

In this work, the formation and performance of LbL membranes and the use of different PCs with their counterions are framed in the context of specific (poly)ion effects. The PCs used have different chaotropic characteristics: (PDADMAC > PAMA-I > PAMA-Cl > PAH). The membranes prepared with PDADMAC show the highest mass adsorption as a result of the low charge availability and more extrinsic charge compensation following the LMWA. This translates into the formation of open layers with the highest PWP between the membranes prepared and low MWCOs. The high mass adsorption brings out a distinct difference between the charged nature of the different terminating BLs (PA and PC), as shown with the zeta potential. This is also shown in the retention of charged solutes (Na_2_SO_4_ and MgCl_2_).

The least chaotropic PAH shows high CD and easily associates with the PA, leading to low mass adsorption thanks to the formation of intrinsic linkages. The tight interaction between PAH and PSS translates into the lowest PWP and MWCO. Moreover, it results in less interdiffusion of the PEs between the layers, as confirmed by the retention of Na_2_SO_4_ and MgCl_2_. Finally, for PAMA-containing membranes, the chaotropicity lays in between PDADMAC and PAH. Moreover, the influence of its counterions (either I^–^ or Cl^–^) and the strength of the interaction PAMA forms with them, give rise to peculiar behavior. Both the PAMA polymers have mass adsorption in between PDADMAC/PSS and PAH/PSS. Between the two, PAMA-I shows the formation of more open layers, lower size exclusion abilities (higher MWCO and lower MgSO_4_ retention). On the contrary, PAMA-Cl forms denser layers and has better size exclusion behavior. Overall, the chaotropicity of PAMA membranes allows for the formation of the right balance between extrinsic and intrinsic charge compensation with PSS. This leads to stable charge exclusion performances regardless of the nature of the terminating layer, unlike the membranes coated with PDADMAC/PSS layers.

Although a more complete evaluation of these effects would still need to address different PAs and include the charge availability in its description, the contextualization of the PEs’ interaction within the specific (poly)ion effects performed in this work already, aids and expands the understanding of the influence of the CD of PCs without ignoring the chemical nature of the functional groups in their monomer units.

## Supporting Information Available

^1^H NMR spectrum of PAMA-I with peak assignment and integration (PDF)
