# Supplementary material for: Addressing Specific (Poly)ion Effects for Layer-by-Layer Membranes
Source: ACS Appl Polym Mater. 2023 Feb 10;5(3):2032–42. doi: 10.1021/acsapm.2c02078 (PMC10012173; doi:10.1021/acsapm.2c02078)

Supporting information

Addressing specific (poly)ion effects for layer-by-layer membranes

Daniëlle Scheepers+, Anna Casimiro+, Zandrie Borneman, Kitty Nijmeijer*

Membrane Materials and Processes, Department of Chemical Engineering and Chemistry, Eindhoven University of Technology, P.O. Box 513, 5600 MB, Eindhoven, The Netherlands.

+Both authors contributed equally to the work.

*Corresponding author: d.c.nijmeijer@tue.nl

Table of Contents

1. ^{1}H NMR of PAMA-I with integration..1

1. ^{1}H NMR of PAMA-I with integration

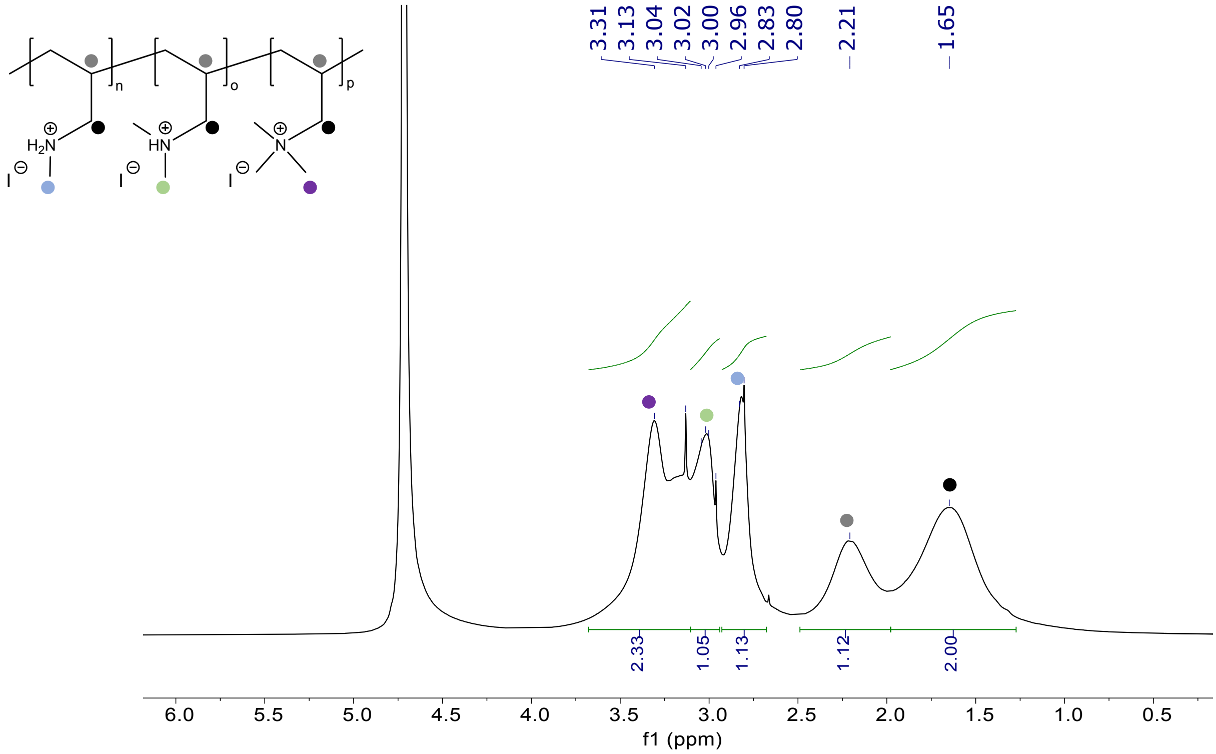

Supplement: Supplementary file 1 — ap2c02078_si_001.pdf [file ap2c02078_si_001.pdf]
